# Paraptosis—A Distinct Pathway to Cell Death

**DOI:** 10.3390/ijms252111478

**Published:** 2024-10-25

**Authors:** Claudia Kunst, Deniz Tümen, Martha Ernst, Hauke Christian Tews, Martina Müller, Karsten Gülow

**Affiliations:** Department of Internal Medicine I, Gastroenterology, Hepatology, Endocrinology, Rheumatology, Immunology, and Infectious Diseases, University Hospital Regensburg, 93053 Regensburg, Germany; deniz.tuemen@ukr.de (D.T.); martha.ernst@stud.uni-regensburg.de (M.E.); hauke.tews@ukr.de (H.C.T.); martina.mueller-schilling@ukr.de (M.M.); karsten.guelow@ukr.de (K.G.)

**Keywords:** paraptosis, cell death, endoplasmic reticulum stress, cytoplasmic vacuolation, reactive oxygen species (ROS), anticancer therapy

## Abstract

Cell death is a critical biological process necessary for development, tissue maintenance, and defense against diseases. To date, more than 20 forms of cell death have been identified, each defined by unique molecular pathways. Understanding these different forms of cell death is essential for investigating the pathogenesis of diseases such as cancer, neurodegenerative disorders, and autoimmune conditions and developing appropriate therapies. Paraptosis is a distinct form of regulated cell death characterized by cytoplasmic vacuolation and dilatation of cellular organelles like the mitochondria and endoplasmic reticulum (ER). It is regulated by several signaling pathways, for instance, those associated with ER stress, calcium overload, oxidative stress, and specific cascades such as insulin-like growth factor I receptor (IGF-IR) and its downstream signaling pathways comprising mitogen-activated protein kinases (MAPKs) and Jun N-terminal kinase (JNK). Paraptosis has been observed in diverse biological contexts, including development and cellular stress responses in neuronal, retinal, endothelial, and muscle cells. The induction of paraptosis is increasingly important in anticancer therapy, as it targets non-apoptotic stress responses in tumor cells, which can be utilized to induce cell death. This approach enhances treatment efficacy and addresses drug resistance, particularly in cases where cancer cells are resistant to apoptosis. Combining paraptosis-inducing agents with traditional therapies holds promise for enhancing treatment efficacy and overcoming drug resistance, suggesting a valuable strategy in anticancer therapy.

## 1. Introduction: Mechanisms of Cell Death

Cell death is a fundamental process in biology, essential for the development, maintenance, and health of multicellular organisms. It plays a central role in shaping tissues during development, maintaining tissue homeostasis, and defending against disease. As stated by Krammer et al., there is “No life without death”; the process of cell death is vital for the life of higher eukaryotes [1]. The paradigm has long been that there are two types of cell death [2]: necrosis, an uncontrolled form in which the cell bursts, releasing its contents and triggering inflammation and apoptosis, a controlled process in which the cell disintegrates into so-called apoptotic bodies while maintaining the integrity of the plasma membrane without causing inflammation. Today, we know that in addition to apoptosis, there are a variety of different subtypes of cell death, each of which is regulated and characterized by its own finely tuned control mechanisms [3]. Still, the field continues to grow, and new mechanisms coordinating multiple cell death pathways are continuously discovered. Thus, up until today, at least 20 different forms of cell death have been described and classified, each distinguished by its specific molecular processes [2,3,4].

### 1.1. Cell Death Classification by the Types of Morphological Alterations: Apoptosis, Autophagy, Necrosis

Cell death is accompanied by visible changes in morphology. Historically, these morphological features have been used to categorize cell death into three distinct forms [4]:

#### 1.1.1. Type I Cell death—Apoptosis

Apoptosis is a tightly regulated, energy-dependent form of programmed cell death that occurs without triggering inflammation. Morphological changes include cell and nuclear shrinkage, chromatin condensation, and the fragmentation of DNA and the cell. The cell fragments into membrane-bound apoptotic bodies, preventing the release of cytosol and cellular components, thus avoiding inflammation. These apoptotic bodies are phagocytosed by immune cells [1,5,6,7].

**Figure 1:**
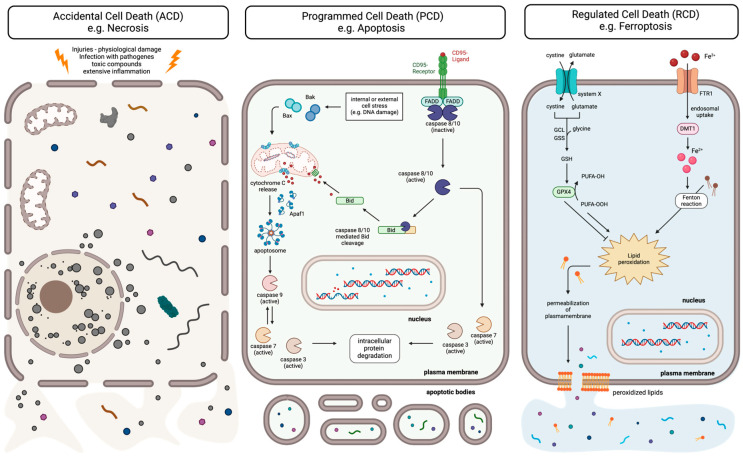
Types of cell death. Accidental cell death (ACD), e.g., necrosis, occurs due to acute and severe physical or chemical injury and is not controlled or regulated by defined mechanisms. Programmed cell death (PCD), e.g., apoptosis, is a highly regulated and controlled process of cellular self-destruction occurring under physiological conditions. PCD, thereby, eliminates unwanted or damaged cells without causing inflammation. Regulated cell death (RCD), e.g., ferroptosis, is a controlled process of cellular demise mediated by specific molecular pathways and mechanisms that can be initiated, regulated, and executed under specific conditions. Left panel: Necrosis is an uncontrolled process in which rupture of the plasma membrane occurs, leading to the release of cellular components and triggering an inflammatory response. Middle panel: In apoptotic cell death, two signaling pathways exist. The extrinsic pathway is initiated by the stimulation of a death receptor (such as CD95, FAS, and APO-1) by its natural ligand. Upon stimulation, the death receptor oligomerizes, forming the death-inducing signaling complex (DISC). At the DISC, initiator caspases are activated, which, in turn, activate effector caspases that initiate apoptosis. In parallel, there is the intrinsic pathway, in which pro-apoptotic molecules are released from the mitochondria, leading to the formation of a cytosolic death platform known as the apoptosome. At the apoptosome, initiator caspases are activated, which then activate effector caspases to proceed with apoptosis. Both pathways can communicate with each other: initiator caspases activated at the DISC can cleave the pro-apoptotic Bcl-2 family member Bid. Truncated Bid (tBid) then induces the intrinsic apoptotic pathway. During apoptosis, no cellular components are released. Instead, apoptotic bodies are formed, which are subsequently taken up by phagocytic cells. This process does not trigger an inflammatory response. Right panel: ferroptosis involves lipid peroxidation. Normally, lipid peroxidation is prevented by the precise regulation and binding of iron molecules, as well as by glutathione peroxidase 4 (GPX4). As a result, iron is present in the cell only in its bound form, not free. GPX4 requires reduced glutathione (GSH) as a cofactor. The availability of cysteine and reduced glutathione (GSH) is regulated by the cystine–glutamate antiporter system Xc—or the transsulfuration pathway. If there is a malfunction in these mechanisms, iron can be released, leading to the production of hydroxyl radicals, which, in turn, cause lipid peroxidation. GPX4 may prevent this process. If the GPX4 function is also impaired, cellular membranes, including the plasma membrane, are severely damaged, and cytosolic components are released. This form of cell death, similar to necrosis, also triggers inflammatory responses. This figure was created with the assistance of BioRender.com (accessed on 19 September 2024).

Apoptosis is induced via two main pathways: the intrinsic pathway, initiated by internal stress such as DNA damage, and the extrinsic pathway, activated by the binding of death ligands to cell surface death receptors. Both pathways activate initiator caspases (caspase-2, -8, -9, -10), which, in turn, activate effector caspases (caspase-3, -6, -7) that drive cell death (Figure 1; middle panel) [1,5,6].

Regarding the intrinsic apoptotic pathway, cellular stress caused by damage to macromolecules such as nucleic acids, proteins, or lipids triggers the release of mitochondrial proteins, including cytochrome c and Smac/DIABLO, into the cytoplasm. The balance between pro- and anti-apoptotic Bcl-2 proteins plays a critical role in the release of these proteins. Stress sensors, such as the tumor suppressor p53 [8,9], are activated by this macromolecular damage and shift the balance in favor of pro-apoptotic Bcl-2 family members. Consequently, the pro-apoptotic Bcl-2 proteins BAX (Bcl-2-associated X protein) and BAK (Bcl-2 homologous antagonist/killer) form pores in the outer mitochondrial membrane. In the cytosol, cytochrome c binds to dATP, Apaf-1, and caspase-9 to form the death platform called apoptosome, where caspase-9 is subsequently activated. Caspase-9 then activates downstream effector caspases, such as caspase-3, caspase 6, and caspase-7, which cleave so-called death substrates and drive the final stages of apoptosis, leading to cell death [7,10,11,12,13]. 

The extrinsic apoptotic pathway is initiated by the binding of ligands to receptors of the tumor necrosis factor (TNF) receptor superfamily, which includes tumor necrosis factor receptor 1 (TNFR1/DR1), CD95 (Fas/Apo-1), death receptor (DR3), TRAIL Receptor 1 (DR4), TRAIL Receptor 2 (DR5), death receptor 6 (DR6), Ectodysplasin A Receptor (EDAR), and nerve growth factor receptor (NGFR) [14,15]. These death receptors are expressed on many cell types and are subject to limited regulation [16,17,18], while their ligands (TNF, CD95L, and TRAIL) are tightly regulated [1,14,19,20]. Ligand binding induces receptor oligomerization and the formation of a death-inducing signaling complex (DISC). This complex is assembled via the recruitment of adaptor proteins like the FAS-associated death domain protein (FADD) and the tumor necrosis factor receptor type 1-associated DEATH domain protein (TRADD), along with initiator caspases-8 and -10 and regulatory proteins like the FLICE-like inhibitory protein (cFlip). Once activated at the DISC, these caspases cleave and activate effector caspases (caspase-3, -6, and -7), which then execute apoptosis [14,21,22,23].

#### 1.1.2. Type II Cell Death—Autophagy

Autophagy is a vital physiological process that transports cytoplasmic components to the lysosome for breakdown, playing a crucial role in cell survival during stress conditions [24,25]. When autophagy is inhibited, cells are transferred into necrosis [26]. Although blocking autophagy has a limited impact on cell death, it increases the occurrence of cellular debris, which is associated with necroptosis [27]. This implies that autophagy activation during necroptosis helps degrade cellular debris rather than inducing cell death [28]. Defects in autophagy or lysosomal integrity are linked to neurodegenerative diseases associated with aging [29].

During autophagy, the phagophore encloses proteins or organelles destined for degradation, forming an autophagosome. In mammalian cells, the autophagosome fuses with lysosomes, while in yeast and plant cells, it merges with vacuoles, where its contents are degraded [25,30,31,32]. A specific form of autophagy is mitophagy, which serves to remove damaged mitochondria. Damage to mitochondria, particularly to the electron transport chain, can lead to the release of reactive oxygen species (ROS), which can oxidize macromolecules such as nucleic acids, proteins, and lipids, potentially causing significant cellular damage [33,34].

#### 1.1.3. Type III Cell Death—Necrosis

Both apoptosis and autophagy are recognized as highly regulated processes. In contrast, necrosis is considered an uncontrolled form of cell death caused by external factors, such as hypoxia or inflammation. Unlike apoptosis, necrosis does not require energy and results from severe cell damage due to stressors such as radiation, heat, or chemicals. This damage then leads to cell swelling and eventually rupture, releasing cellular contents into the surrounding tissue, which triggers inflammation and tissue injury (Figure 1, left panel). Thus, necrosis often involves an increase in pro-inflammatory proteins like nuclear factor-κB [2,6,35].

As more forms of cell death were gradually identified, this simple classification became inadequate. Therefore, in 2005, the Nomenclature Committee on Cell Death introduced a nomenclature that reflects these developments. It highlighted the need for precise identification and distinction between different cell death types, underscoring the significance of molecular pathways, genetic influences, biochemical indicators, and functional parameters. This nomenclature was gradually expanded thereafter [4,36,37,38,39]. Given the rapid ongoing progress in this field, in 2018, the Nomenclature Committee on Cell Death suggested a revised classification of cell death, emphasizing the mechanistic and essential aspects of each cell death process [4]. 

### 1.2. Cell Death Classification by the Type of Regulation: Accidental, Regulated, and Programmed Cell Death 

#### 1.2.1. Accidental Cell Death (ACD)

Accidental cell death refers to a form of cell demise that occurs due to acute and severe physical or chemical injury rather than by regulated or programmed mechanisms. It typically results from extreme stress or trauma that overwhelms the capacity of the cell to maintain homeostasis and survival. Unlike programmed cell death, ACD is not controlled or orchestrated by specific molecular pathways and may contribute to tissue damage and inflammation in pathological conditions. Thus, ACD constitutes a passive process, with unregulated necrosis being the major type (Figure 1, left panel) [4,40].

#### 1.2.2. Programmed Cell Death (PCD) 

Programmed cell death refers to a regulated and controlled process of cellular self-destruction that occurs under physiological conditions, i.e., as a normal part of development, homeostasis, or in response to specific signals. It is characterized by distinct biochemical and morphological changes, such as cell shrinkage, chromatin condensation, and fragmentation into apoptotic bodies. Programmed cell death serves important functions in eliminating unwanted or damaged cells without causing inflammation or harm to the surrounding tissues. Therefore, PCD represents a purely physiological form of RCD [3,4,41].

Among the best-characterized forms of PCD is the aforementioned apoptosis (Figure 1, middle panel) when occurring in a physiologic context, e.g., intestinal epithelial cells, which are naturally shed into the lumen as part of their regular lifecycle, undergo anoikis (a special form of apoptosis induced by loss of cell anchorage) to maintain tissue homeostasis and barrier function [42,43]. Also, necroptosis can be classified as PCD when it occurs in a physiological context. Necroptosis is a strictly regulated cellular suicide program that is activated when apoptosis is inhibited. Mechanistically, it is similar to apoptosis, but morphologically, it is similar to necrosis [44]. Necroptosis can be induced by several stimuli, including the activation of death receptors or Toll-like receptors. When caspase-8 is inhibited at the DISC, receptor-interacting serine/threonine kinase 1 (RIP1) can be stabilized. RIP1 then phosphorylates and activates RIP3, which, in turn, phosphorylates mixed lineage kinase domain-like pseudokinase (MLKL). The phosphorylation of MLKL induces a conformational change and allows MLKL to bind to inositol hexaphosphate, which then leads to the recruitment of MLKL to phosphatidylinositides and its insertion into the plasma membrane. At this site, MLKL oligomers form, resulting in permeabilization of the plasma membrane. As a consequence, cytosolic contents are released, resulting in cell death [41,45,46,47,48,49,50,51].

#### 1.2.3. Regulated Cell Death (RCD) 

Regulated cell death refers to a controlled and orderly process of cellular demise that is mediated by specific molecular pathways and mechanisms. Unlike ACD, RCD involves signaling pathways that can be initiated, regulated, and executed under specific physiological or pathological conditions. Moreover, RCD depends on specific cellular signaling mechanisms, which can be influenced by pharmacological or genetic approaches [4,41]. 

A well-characterized form of RCD is ferroptosis [4,52,53,54], which is triggered by iron-mediated lipid peroxidation [52,53,54,55]. ROS, primarily generated in the mitochondria as byproducts of cellular respiration [56,57], can oxidize macromolecules like nucleic acids, proteins, and lipids. Among these ROS is hydrogen peroxide (H_2_O_2_), which reacts with free iron (Fe^2+^) in the Fenton reaction, producing highly reactive hydroxyl radicals that induce lipid peroxidation, ultimately leading to cell death [58]. Initially thought to be a form of necrosis [58], ferroptosis is now recognized as a regulated process crucial in neurodegeneration and cancer, such as liver, breast, and lung cancer [59,60,61,62]. Ferroptosis can be inhibited by glutathione peroxidase 4 (GPX4), which converts lipid peroxides into non-toxic lipid alcohols, or by FSP1, which reduces non-mitochondrial coenzyme Q10 to generate an antioxidant that prevents lipid peroxidation. Morphologically, ferroptosis is marked by cell membrane rupture, increased membrane density, mitochondrial atrophy, the loss of cristae, and chromatin condensation (Figure 1, right panel) [54,63,64,65]. 

Notably, it is becoming increasingly evident that RCD pathways do not function in an isolated manner. Instead, they are part of a complex, interconnected network where multiple RCD and PCD pathways share components and signaling mechanisms [66,67]. This extensive crosstalk between different forms of cell death highlights the existence of a vast cell death network within cells. Through these overlapping pathways, cells maintain multiple backup mechanisms to ensure the execution of cell death, underscoring the intricate balance and redundancy built into these processes.

Understanding and differentiating between the many different forms of cell death and their control mechanisms is crucial for assessing the pathogenesis of diseases like cancer, neurodegenerative disorders, and autoimmune conditions [2].

## 2. Morphological and Molecular Characteristics of Paraptosis

While well-characterized forms of cell death like apoptosis and necrosis have been extensively studied, emerging research has identified alternative cell death mechanisms that are less understood but equally significant. One such pathway is paraptosis, a distinct form of non-apoptotic cell death that is gaining attention for its potential role in cancer therapy and resistance.

In 2000, Sperandio et al. were the first to describe this type of cell death, which differs from apoptosis in terms of morphology, biochemistry, and the response to specific inhibitors of apoptosis [68]. It was titled “paraptosis” (derived from the Greek suffix para (= next to/related to) and apoptosis). At that time, only necrosis, apoptosis, autophagy, lysosomal cell death, and mitoptosis were known forms of cell death [2]. Today, according to the classification system of the Nomenclature Committee on Cell Death, paraptosis is considered a form of RCD. 

**Figure 2:**
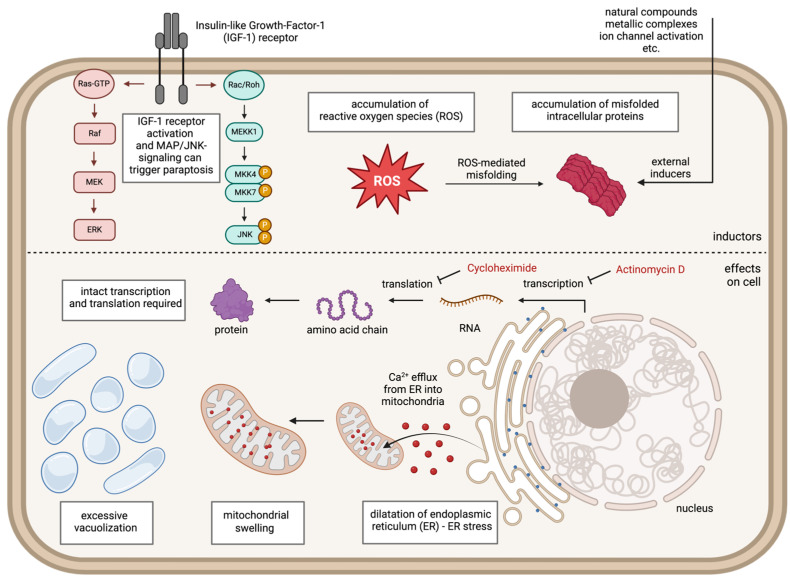
Characteristics of paraptosis. Paraptosis is a form of regulated cell death that significantly differs from apoptosis in terms of morphology, biochemistry, and response to specific inhibitors. Paraptosis can be triggered by IGF-IR-dependent signaling in its downstream signaling pathways, including mitogen-activated protein kinases (MAPKs) and Jun N-terminal kinase (JNK), reactive oxygen species (ROS)-mediated cellular damage or the accumulation of misfolded proteins. These triggers lead to a dilatation of the endoplasmic reticulum (ER), creating osmotic pressure that draws water from the cytoplasm. Additionally, a Ca^2+^ flux between the ER and mitochondria through the ER–mitochondrial axis leads to mitochondrial swelling from Ca^2+^ overload. Vacuoles can be derived from both dilatated ER and mitochondria. In the final step, these processes lead to the loss of plasma membrane integrity, resulting in cell death. Thus, morphologic hallmarks of paraptosis are the accumulation of large fluid-filled cytoplasmic vacuoles and the dilatation of both ER and mitochondria. The process of paraptosis requires de novo protein synthesis and can be blocked by inhibitors of transcription (e.g., Actinomycin D) and translation (e.g., Cycloheximide). This figure was created with the assistance of BioRender.com (accessed on 19 September 2024).

Paraptotic cell death is characterized by specific morphologic alterations such as cytoplasmic vacuolation and the dilatation of essential cellular components such as mitochondria and the endoplasmic reticulum (ER). This is followed by the loss of plasma membrane integrity, which ultimately leads to cell death. Depending on the specific trigger inducing paraptosis, different signaling molecules may be involved in its regulation. The main characteristics of paraptosis are illustrated in Figure 2. 

### 2.1. Cytoplasmic Vacuolation

Morphologically, paraptosis is characterized by chromatin condensation, excessive cytoplasmic vacuolation, and, in the late phase, the swelling of mitochondria, which also lose the structure of their cristae. In the initial description of paraptosis, cytoplasmic vacuolation was detected both by light microscopy and electron microscopy [68]. It is especially the accumulation of these large fluid-filled vesicles with single membranes that is used for the detection of paraptosis [68,69]. In contrast to apoptosis, apoptotic bodies, as well as DNA fragmentation or condensation, are absent.

### 2.2. Dilatation of the Endoplasmic Reticulum and Mitochondria

The characteristic formation of cytoplasmic vesicles during paraptosis is due to the dilatation of both the ER and mitochondria [68,70]. In a study of curcumin-induced paraptosis in human glioblastoma cells, these vesicles were even shown to have ribosomes attached to their membranes, indicating their ER origin [71]. ER expansion results from the accumulation of misfolded proteins in the ER lumen, driving osmotic pressure to draw water from the cytoplasm [2,72,73]. In addition, communication between the ER and mitochondria occurs via a Ca^2+^-flux mechanism during paraptosis. ER stress and subsequent dilatation can lead to the release of Ca^2+^ from the ER to the mitochondria via the so-called ER–mitochondrial axis. This Ca^2+^-flux may then cause a mitochondrial Ca^2+^ overload and, thus, mitochondrial swelling [2,73]. 

These morphological changes characteristic of paraptosis can be induced by different mechanisms, including disruptions in the regulation and maintenance of the cellular protein environment, e.g., by ROS, inhibition of the proteasome, impaired protein thiol balance (the maintenance of the redox state of thiol groups and disulfide bonds within proteins, which are essential for protein folding during stability, redox signaling, and cellular defense [74]), and imbalanced ion homeostasis [73,75,76,77,78]. ER stress and Ca^2+^ overload can also be promoted by ROS [78]. Thioredoxin reductase 1, an important component of the antioxidant thioredoxin system, can prevent paraptosis by reducing ROS levels and reverting thiol oxidation [78,79]. Consistently, the inhibition of thioredoxin reductase 1 has been shown to enhance paraptotic cell death in glioblastoma multiforme cells [80]. 

### 2.3. Requirement of Transcription and Protein Synthesis

On the molecular level, paraptosis requires intact protein synthesis and can be blocked by transcription inhibitors like actinomycin D and translation inhibitors like cycloheximide. In contrast, the inhibition of caspases is ineffective at blocking paraptotic cell death since the apoptotic caspase cascade is not involved [2,68,81]. 

### 2.4. Signaling via IGF-IR, MAPK, and JNK

Sperandio et al. demonstrated that paraptosis could be triggered by the insulin-like growth factor I receptor (IGF-IR) and its downstream signaling pathways, including mitogen-activated protein kinases (MAPKs) and Jun N-terminal kinase (JNK) pathways in 293T cells. IGF-IR-induced paraptosis is suppressed by inhibitors specific to the dual specificity MAPK kinase 2 (MEK-2) and by antisense oligonucleotides targeting c-Jun N-terminal kinase-1 (JNK-1). The endogenous counteraction of paraptosis is mediated by AIP-1/Alix (apoptosis-linked gene 2 (ALG-2)-interacting protein 1/ALG-2 and interacting protein X), a multifunctional protein, which has been shown to interact with cell death-related calcium-binding protein ALG-2 [82,83] and to be involved in cell death regulation, vesicle trafficking, and viral budding [84]. Notably, AIP-1/Alix specifically inhibits paraptosis without affecting apoptosis [81]. Another study using 293T cells revealed that overexpression of TAJ/TROY, a member of the tumor necrosis factor receptor superfamily, which is abundantly expressed during embryonic development [85], triggers non-apoptotic cell death with paraptosis-like characteristics [86].

Noteworthy, paraptosis involves caspase-9 activity, which functions independently of its apoptotic role, being Apaf-1-independent, insensitive to caspase inhibitors, and independent of processing from its precursor form [3,68]. Thus, caspase-9 plays a key role in both apoptotic and non-apoptotic cell death, particularly in IGF-IR-induced paraptosis. Caspase-9 co-immunoprecipitated with IGF-IR and a dominant-negative mutant of caspase-9, unlike other caspases, blocked paraptotic cell death. Inhibiting apoptosis with the broad-spectrum caspase inhibitor BAF revealed that caspase-9 also induces paraptosis, mimicking IGF-IR effects. Mutations blocking caspase-9 processing reduced apoptosis while increasing paraptosis, emphasizing its dual function in regulating both cell death types [68].

### 2.5. Molecular Modulators

Several molecular modulators have been identified to be involved in paraptosis regulation. Proteomic analysis of 293T cells undergoing paraptosis revealed changes in the amount or subcellular localization of structural, signaling, metabolic, and mitochondrial proteins [87]. Due to the morphologic changes in paraptosis, the levels of certain cytoskeletal proteins are altered. For example, β-tubulin levels were reduced in paraptotic cells, whereas α-tubulin and tropomyosin are redistributed within the cell [87]. These changes reflect the structural rearrangements of the cell during paraptosis, with the early disruption of the microtubular network likely playing a central role. Moreover, increased levels of ATP synthase β-subunit indicate that energy is required for paraptosis [87]. Phosphatidylethanolamine binding protein-1 (PEBP-1) has been identified as a modulator of paraptosis that is reduced during this type of cell death [87]. This signaling protein is also known as RKIP (Raf kinase inhibitor protein) and is involved in the regulation of a variety of other signaling pathways. It inhibits the Raf/MEK/ERK signaling cascade, thus playing a crucial role in cell differentiation, proliferation, and other forms of cell death, such as apoptosis and ferroptosis [88,89,90]. PEBP-1 also modulates pathways related to inflammation and metastasis suppression [88,91,92,93,94,95]. PEBP-1 downregulation is essential for paraptosis, as its overexpression prevents IGF-IR-mediated paraptotic cell death [87]. As a second important modulator of paraptosis, the protein prohibitin was identified [87]. Prohibitin is a multifunctional protein involved in regulating cell cycle progression, mitochondrial function, and apoptosis. It acts as a molecular chaperone and is implicated in cellular aging and tumor suppression [96,97]. Prohibitin is localized at the inner mitochondrial membrane or translocates there in response to specific stimuli [87]. Moreover, there is a functional link between prohibitin and p53 activity [98,99], potentially affecting the decision between cell growth and programmed cell death [98,100]. 

Reflecting the involvement of ER dilatation, ER stress markers such as the Binding Immunoglobulin Protein (BiP) and the C/EBP Homologous Protein (CHOP) have been shown to be associated with paraptosis. BiP is a chaperone protein in the ER that supports protein folding. It functions as a sensor for ER stress and can initiate an unfolded protein response (UPR) [101,102]. CHOP is a transcription factor induced by ER stress, promoting cell death when the stress is prolonged or unresolved. Both proteins were induced during paraptosis, and this process was blocked by cycloheximide [103,104]. Consistently, the deletion or inhibition of PERK (pancreatic ER kinase (PKR)-like ER kinase), a key mediator of the UPR that inhibits translation by phosphorylating the initiation factor 2 alpha (eIF2α) in response to an accumulation of misfolded proteins, induced paraptosis in cancer cells [105].

Of note, the protein LC3B (microtubule-associated protein 1 light chain 3 beta), typically recognized as an autophagy marker, also seems to be involved in paraptotic processes. The upregulation and processing of LC3B were also recognized as important events in nonautophagic cytoplasmic vacuolation and cell death since a knockdown conferred significant protection against paraptosis-like cell death in HCT116 cells, a colon carcinoma cell line [106]. Moreover, induction of LC3B has been observed in several cancer cells in response to paraptosis-inducing agents [107,108].

Especially in the context of inducing paraptosis in tumor cells (see Section 2.4 and 4), several additional molecular mediators and modulators were identified as being involved in this form of cell death.

ROS-induced paraptosis in rat T9 glioma cells was associated with the release of “danger signals” like the induction of heat shock proteins and the relocation of HMGB1 (High Mobility Group Box 1) from the nucleus to the cell periphery, thereby potentially enhancing tumor immunogenicity [109]. In response to ginsenoside Rh2, a bioactive compound in ginseng, lung cancer cells upregulated c-Myc, leading to the accumulation of so-called aggresomes containing tribbles homolog 3 (TRIB3) and p62, thereby triggering paraptosis [110].

Another study revealed that ginsenoside Rh2 induces both apoptosis and paraptosis-like cell death in colorectal cancer cells via p53 activation. In the absence of p53, Rh2-induced cell death and vacuole formation were significantly reduced, highlighting the importance of p53 in both processes [111]. 

SHP-2 (Src homology region 2-containing protein tyrosine phosphatase 2), a protein tyrosine phosphatase, plays a critical role in cellular signaling pathways. Recently, Li et al. demonstrated that SHP-2 is an upstream mediator of elaiophylin-induced paraptosis [112]. It acts upstream of the MAPK pathway and has been shown to be a direct target of the natural antibiotic and antiparasitic compound elaiophylin in ovarian cancer cells [112]. USP10 (ubiquitin-specific peptidase 10), a deubiquitinating enzyme that plays a critical role in different cellular processes by regulating the removal of ubiquitin from target proteins, has been shown to be involved in the regulation of curcumin-induced paraptosis in breast cancer cells [113]. Since paraptosis involves a dysregulated Ca^2+^-balance [73], ion channels associated with the homeostasis of intracellular ion transport, have been shown to impair the activation of paraptosis [114,115].

Thus, paraptosis is a cellular stress reaction in response to ER stress, Ca^2+^ overload, the accumulation of misfolded proteins, ionic imbalance, ROS-mediated oxidative stress, or activation of signaling cascades involving IGF-IR, MAPK, and JNK. Depending on the specific stressor, a set of other modulators may be involved. 

### 2.6. Paraptosis and the Network of RCD

The processes outlined above, including ER stress, Ca^2+^ overload, the accumulation of misfolded proteins, ionic imbalance, ROS-mediated oxidative stress, and activation of signaling cascades, such as IGF-IR, MAPK, and JNK, are key factors in paraptosis. However, these mechanisms are not exclusive to paraptosis. Rather, they play a role in multiple forms of cell death, highlighting the interconnectivity of these pathways [66,116,117]. Thus, it is not surprising that paraptosis is often observed alongside other forms of cell death, such as autophagy [118,119], cuproptosis [120,121], and apoptosis [120,121,122,123,124,125,126]. This intricate network of cell death pathways has gained increasing attention. In this regard, understanding how cell fate is determined and directed towards paraptosis would be of significant interest. It has been proposed that different cell death pathways can be triggered within the same cell, with the prevailing death phenotype being dictated by the relative speed at which these programs unfold [111,127,128]. Given this complexity, to date, no comprehensive marker [119] or standardized biochemical assay [113,129,130] exists to specifically detect paraptosis.

In summary, the interplay of specific intracellular stress responses and signaling pathways underlines the complex mechanisms of paraptosis and its relationship with other forms of RCD. Future research should uncover the nuances of these interconnected pathways and determine how cellular fate is dictated, which is essential for developing targeted therapeutic strategies. Additionally, identifying specific biomarkers and refining detection methodologies for paraptosis could pave the way for innovative cancer treatments that exploit this unique form of cell death.

## 3. (Patho)physiological Function of Paraptosis

**Figure 3:**
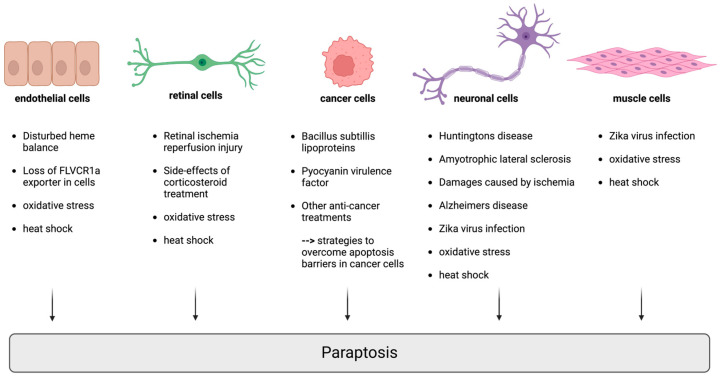
Occurrence of paraptosis under physiological and pathophysiological conditions. Paraptosis has been observed in a variety of cell types under both physiological and pathophysiological conditions. Whereas, in healthy cells, paraptosis is rare, this type of cell death can be induced in response to specific stress signals or disrupted cellular homeostasis. This figure was created with the assistance of BioRender.com (accessed on 19 September 2024).

Paraptosis has been observed in a variety of (patho)physiological contexts (Figure 3). These include the following:

### 3.1. Developmental Processes

Paraptosis has been implicated in normal developmental processes, such as during the differentiation and remodeling of tissues. Furthermore, paraptosis-like cell death has been described to be involved in follicular atresia, the process by which immature ovarian follicles degenerate and are reabsorbed, typically occurring during ovarian development and the menstrual cycle. This study observed dilatated ER in granulosa cells of adult Wistar rats, which were negative for the TUNEL assay and active caspase-3, indicating the distension is not apoptotic or autophagic [131].

### 3.2. Neuronal Cells

Certain neuronal cells may undergo paraptosis under specific conditions. This has been noted in the development, maintenance, and pathological conditions of the nervous system. Neuronal cell death in neurodegenerative diseases such as Huntington’s disease and amyotrophic lateral sclerosis does not meet the criteria for apoptosis but shows characteristics of paraptosis [132]. Moreover, paraptosis is increasingly being recognized as a major contributor to damage caused by ischemia. Alongside autophagy, paraptosis was detected after retinal ischemia–reperfusion injury in an experimental model of acute hypertensive glaucoma, an eye condition damaging the optic nerve and one of the leading causes of blindness [119]. Electron microscopy analysis identified significant cytoplasmic vacuolation within 6 h following retinal ischemia–reperfusion injury and the subsequent death of retinal ganglion cells. The observed vacuoles mainly originated from the progressive swelling of the ER and/or mitochondria in retinal ganglion cells after ischemia–reperfusion injury [119]. Moreover, in a mouse model of Alzheimer’s disease, neurodegeneration was associated with increased cell death rates resembling both paraptosis and autophagy [118]. In this mouse model, the overexpression of the Δ40 isoform of p53 combined with the humanized form of the amyloid precursor protein led to enhanced IGF-1R signaling and increased phosphorylation of MAPK and JNK [118,133,134], which is consistent with the initial description of paraptosis [68,81]. Recently, paraptosis resembling cell death was described in the context of Zika virus infection [125]. The Zika virus is a mosquito-borne flavivirus that causes Zika fever, which can lead to serious birth defects if contracted during pregnancy. It is primarily transmitted through the bite of infected Aedes mosquitoes but can also spread via sexual contact, blood transfusion, and from the mother to the fetus. Infection leads to serious congenital brain defects in the fetus. Human neural progenitor cells, which are particularly susceptible to Zika virus infection, experience disrupted cell proliferation and undergo cell death upon infection [125]. Both apoptotic and non-apoptotic mechanisms play a role in this process. The virus utilizes the ER to assemble replication complexes, causing ER stress and triggering the UPR. Extended ER stress due to the Zika virus infection leads to paraptosis-like cell death, identified by the presence of ER-derived vacuoles in the cytoplasm [124,125], which could be blocked by heparin in human neural progenitor cells, allowing for their differentiation into neuroglia cells [125]. Except for some studies evaluating the paraptotic effects of Singapore grouper iridovirus on fish cells [135], there is limited data in the literature on virus-induced paraptosis.

### 3.3. Retinal Cells

Retinal cell death induced by corticosteroids is a side effect of corticoid therapy. A study on rats and retinal cells from rat and human origins indicated that paraptosis might be the underlying mechanism of ocular toxicity of corticosteroids [136,137].

### 3.4. Endothelial Cells

A disturbed heme balance has been shown to induce paraptosis in endothelial cells. Heme is essential for cellular respiration and survival. However, intracellular heme levels must be tightly controlled to prevent excess, leading to ROS generation and subsequent cell death. The loss of the heme exporter FLVCR1a (Feline Leukemia Virus sub-group C Receptor 1a) disrupts heme balance in endothelial cells, causing impaired angiogenesis and cell death via paraptosis, with ER stress occurring before paraptotic cell death [138]. These data underscore the physiological role of paraptosis in the regulation of intracellular heme, endothelial cell homeostasis, and angiogenesis.

### 3.5. Muscle Cells, Epithelial Cells, Fibroblasts, and Astrocytes

Paraptosis can occur in muscle cells under specific circumstances, contributing to tissue remodeling and stress responses. Similar to the effects seen in human neuronal progenitor cells [125], the Zika virus infection induced caspase-independent cell death in human primary myoblasts, which was associated with significant vacuolation along with the development of ER membranes and clusters of vesicles [139]. Consistent cytopathic findings were observed in human epithelial cells, primary skin fibroblasts, and astrocytes. The formation of large ER-derived cytoplasmic vacuoles depended on PI3K/Akt signaling and was followed by a destructive inward collapse of the cell structure, leading to cell death [124].

### 3.6. Response to Cellular Stress

Cells can undergo paraptosis in response to different stressors, such as oxidative stress, heat shock, and certain toxic compounds. This process may serve as a cellular defense mechanism to eliminate damaged cells. In a mouse model of lung inflammation induced by 20 nm amorphous silica nanoparticles, key markers of paraptosis, such as the presence of large vacuoles and the expansion of the ER and mitochondrial swelling, were observed. These findings suggest that paraptosis may play a role in nanomaterial-induced lung inflammation [140]. 

One study demonstrated that iturin A-like Bacillus subtilis lipoproteins induce paraptosis in Caco-2 cells, suggesting their potential as anti-tumor agents [141]. Moreover, pyocyanin, a virulence factor expressed by the biofilm-forming bacterium Pseudomonas aeroginosa, triggered paraptotic effects such as ER dilatation and the formation of ER-derived cytoplasmic vacuoles in renal tubular epithelial cells [142]. Apart from these two studies, there are, to our knowledge, no other reports in the literature describing bacterial-induced paraptosis. However, since bacteria can produce ROS and affect the redox state in their immediate environment [143], it is conceivable that they may also be capable of inducing paraptosis. 

### 3.7. Cancer and Anticancer Treatments

Paraptosis has been observed in cancer cells, particularly in response to certain anticancer treatments. Some therapies aim to induce paraptosis as a means of eliminating cancer cells resistant to apoptosis. The aspect of paraptosis as a therapeutic tool in anticancer therapy will be discussed in detail in the next section. 

## 4. Induction of Paraptosis—An Innovative Strategy in Anticancer Therapy

Paraptosis represents a form of RCD that occurs as a specific response to cellular stress. As discussed in Section 2.3, paraptosis is rare in a healthy organism and primarily arises under pathophysiological conditions when cellular homeostasis is disrupted. The preferred pathway for eliminating damaged or abnormal cells is usually apoptosis. Despite the fact that apoptotic cell death persists to a certain extent in malignant disease and often even indicates poor prognosis, the inhibition or evasion of apoptosis is a well-established oncogenic mechanism in cancer cells [144]. The inhibition of apoptosis enables malignant cells to survive and sustain uncontrolled proliferation. These so-called hallmarks of cancer are fundamental biological capabilities that enable cancer development and progression [145,146]. Many cancer therapies aim to restore or induce apoptosis or other types of cell death to stop tumor progression. Resistance to cell death is a key feature of cancer development associated with treatment failure and, thus, presents a challenge in the clinical management of malignant diseases [147,148]. Therefore, alternative cell death pathways such as paraptosis offer additional therapeutic strategies, complementing treatments that rely on the activation of conventional cell death mechanisms. The relevance of paraptosis in cancer therapy is highlighted by the fact that at least 75% of the PubMed (https://pubmed.ncbi.nlm.nih.gov/ (accessed on 19 September 2024)) search results for “paraptosis” focus on paraptosis in cancer cells. The concept is to use external triggers to disrupt cellular homeostasis by inducing mechanisms such as ER stress, Ca^2+^ overload, the accumulation of misfolded proteins, ionic imbalance, ROS-mediated oxidative stress, or the activation of signaling cascades involving IGF-IR, MAPK, and JNK. These perturbations ultimately induce paraptosis as an alternative or additive stress response.

**Figure 4:**
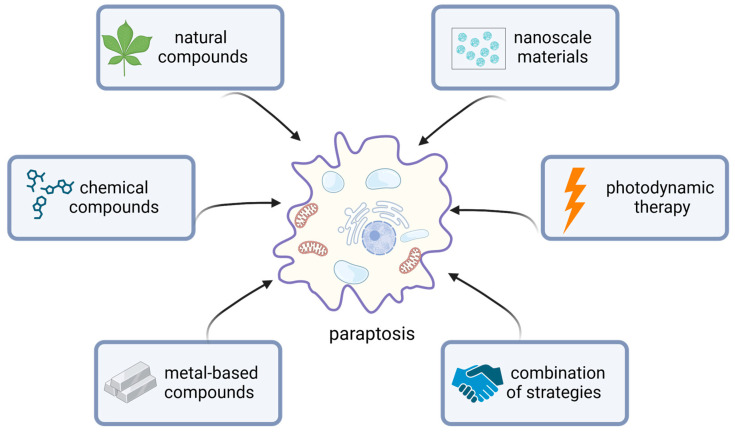
Induction of paraptosis as a therapeutic strategy in anticancer therapy. A variety of compounds have been shown to induce paraptosis in tumor cells. Specific substances can either selectively induce paraptosis or combine it with other cell death modalities. The targeted triggering of these mechanisms can enhance therapeutic efficacy and address resistance to specific treatments. This figure was created with the assistance of BioRender.com (accessed on 19 September 2024).

Growing evidence suggests that several compounds or triggers can induce paraptosis and inhibit cancer cell growth in vitro and in vivo (Figure 4) [3,148].

### 4.1. Natural Compounds

Natural compounds have emerged as promising agents for inducing paraptosis in cancer cells due to their ability to trigger stress responses and disrupt cellular homeostasis. These compounds, often derived from plants, fungi, or marine organisms, can induce paraptosis through mechanisms such as ER stress, oxidative stress, and mitochondrial dysfunction. For example, compounds like ginsenoside Rh2 and curcumin have been shown to activate pathways leading to paraptosis by causing cellular stress responses and interfering with crucial signaling pathways (Table 1). By harnessing these natural compounds, researchers aim to develop novel therapeutic strategies that exploit paraptosis to more effectively target and eliminate cancer cells.

A range of different natural compounds have been shown to induce paraptosis in cancer cells. Some of them have been reviewed in recent articles [3,75,76,148,149,150]. Table 1 highlights a selection of key, paraptosis-inducing natural products as well as those analyzed from 2023 to 2024 and their mechanism of action. Notably, many of these compounds not only induce paraptosis independently but also have the potential to enhance the efficacy of established cancer therapies or restore their efficacy in cases of resistance.

**Table 1:** Natural compounds inducing paraptosis in tumor cells.

Compound	Origin	Type of Tumor Cell	Mechanism	Ref.
Aloperine	*Sophora alopecuroides*	Glioblastoma	ER stress ROS production CHOP induction Activation of MAPK	[151]
Brassinin	cruciferous vegetables	Chronic myelogenous Leukemia	ROS production ER stress Mitochondrial damage Activation of MAPK	[152]
Cannabidiol	*Cannabis sativa*	Colorectal cancer Breast cancer	ER stress ROS production Activation of MAPK	[153,154]
Chalcomoracin	*Morus alba*	Breast cancer Prostate cancer	Alix downregulation, ROS production	[155]
Curcumin	*Curcuma longa*	Malignant breast cancer	Mitochondrial Ca^2+^ overload, Proteasomal dysfunction	[156,157,158]
		Glioblastoma	ER stress, Modulation of Akt-insulin- and p53-Bcl2-networks via regulation of miRNAs	[71]
		Prostate cancer	ER stress, ROS production	[159]
Docosahexaenoic acid	Marine fish	Colorectal cancer	Acts in combination with sodium selenite Disruption of redox homeostasis Activation of MAPK	[160]
Elaiophylin	*Streptomyces melanosporus*	Ovarian cancer	MAPK hyperactivation	[112]
Fangchinoline	*Stephania tetrandra*	Renal cancer	ROS production ER stress	[161]
Ginsenoside Rh2	*Panax ginseng*	Colorectal cancer	Activation of p53- and NF-κB signaling	[111]
		Lung cancer	c-Myc-mediated accumulation of tribbles homolog 3 (TRIB3)/P62(111) aggresomes when combined with everolimus	[110]
α-Hederin	*Akebia trifoliata*	Colorectal cancer	Alix downregulation Activation of MAPK signaling by enhanced Ca^2+^ flux via G-protein-coupled receptors	[162]
Hesperetin	Citrus fruits	Breast cancer	Cytoplasmic vacuolation Alix downregulation ROS production	[163]
Jolkinolide B	*Euphorbia fischeriana*	Bladder cancer	ROS-mediated ER stress Activation of Erk signaling Synergy with mTOR and glutathione peroxidase 4 inhibition	[164,165,166]
Kaempferide	*Mimosa tenuiflora*	Pancreatic cancer	Alix downregulation Induction of ATF4 and CHOP ROS production	[167]
Kuwanon M	*Morus alba*	Lung cancer	ER stress Alix downregulation Activation of MAPK	[168]
Morusin	*Morus alba*	Ovarian cancer	Ca^2+^ overload and dysfunction of mitochondria	[169]
Orphiobolin A	pathogenic *Bipolaris* fungi	Glioblastoma	Blockage of BKCa channel activity	[170]
Osimertinib	Methylindole-aniline-pyrimidine derivative	Glioblastoma	ER stress Accumulation of ubiquitinated proteins Induction of CHOP	[171]
Paris Saponin II	*Paris polyphylla*	Lung cancer	ER stress Activation of JNK signaling Enhances cytotoxicity of cisplatin	[172]
PFAP	*Pleurotus ferulae lanzi*	Lung cancer	ER stress	[173]
Plumbagin	*Plumbago zeylanica*	Breast cancer	ER stress	[174]
		Cervical cancer	Disruption of sulfhydryl homeostasis	
		Lung cancer	Proteasome inhibition	
Rutin Linoleate	Plants	Lung cancer	Cytoplasmic vacuolation Oxidative stress	[175]
6-shogaol	*Zingiber officinale*	Breast cancer	ER stress	[176]
		Lung cancer	Proteasome inhibition	

### 4.2. Chemical Compounds

There are also several chemically synthesized drugs that induce paraptosis. These compounds belong to different chemical classes, including quinolizidines, isoxazoles, chalcones, benzoylhydrazides, and curcumin derivatives, and have been reviewed in the recent literature [148]. Some of these chemical compounds are developed or further refined based on natural compounds. For instance, derivatives of oxazine, initially extracted from marine fungi, are being optimized for enhanced efficacy in inducing paraptosis [177]. This approach combines the advantages of natural compound activity with the precision of chemical modification, aiming to improve potency and specificity against cancer cells. The latest reports on paraptosis-inducing chemical compounds analyzed from 2023 to 2024 and their mechanism of action are shown in Table 2.

### 4.3. Metal-Based Compounds

Paraptosis-inducing metal complexes are a class of compounds used in tumor therapy that utilize metal ions to trigger cell death. These complexes typically involve metal ions such as platinum, ruthenium, iridium, or copper, coordinated with organic ligands to selectively target cancer cells. Copper-based compounds can induce tumor cell death via multiple mechanisms, such as triggering apoptosis via ROS, suppressing angiogenesis, initiating cuproptosis, and causing paraptosis [120,121]. Recently, Cu^2+^ ions were also used to build so-called dual-ion “nano-trap” nanoparticles (see Section 4.4) to induce paraptosis in breast cancer cells [122]. Furthermore, a study using Zinc (II) ions in complexes of Sirtuin 1/2 (SIRT1/2) inhibitors was observed to exchange with copper ions within cells, leading to the generation of redox-active copper complexes. These complexes triggered ROS production and paraptotic cell death [183]. Cyclometalated iridium (III) complexes have been shown to induce ER stress and paraptosis in hepatoma cells [184]. The most recent studies revealed that metal complexes containing rhenium, silver, and gold ions can also effectively induce paraptosis in cancer cells [185,186,187]. By inducing paraptosis, these metal complexes could bypass the resistance mechanisms that often limit the effectiveness of conventional therapies. Their capacity to cause cellular stress and interfere with critical pathways is a promising strategy for overcoming drug resistance.

### 4.4. Strategies Based on Nanomedicine

Nanomedicine in tumor therapy uses nanoscale materials to deliver targeted treatments with high precision. By incorporating nanoparticles that specifically release therapeutic agents into cancer cells, these advanced treatments can induce cell death through mechanisms such as oxidative stress or mitochondrial dysfunction. This strategy not only enhances the specificity of tumor destruction but also addresses therapeutic resistance, providing a novel avenue for effective cancer treatment. Recent developments focus on paraptosis-inducing nanomedicine to overcome drug resistance. Notable examples include amphiphilic 8-hydroxyquinoline (HQ) block copolymers forming Cu(HQ)_2_ complexes that inhibit proteasomes and induce paraptosis [188] and silver nanoparticles (AgNP) that inhibit pancreatic ductal adenocarcinoma by inducing paraptosis through various cellular disruptions [107]. Additionally, nanosized Cu^2+^-coordinated morusin/doxorubicin combinations—involving a metal ion, the natural compound morusin, and a chemotherapeutic—and Cu(diethyldithiocarbamate)_2_ nanoparticles have shown effectiveness in inducing paraptosis and targeting resistance in tumor cells [189,190]. Recent approaches have used bioactive gallium sulfide nanodots for tumor therapy that reprogram iron metabolism and interfere with iron pathways, thereby inducing a hybrid paraptosis–apoptosis response in cancer cells [123]. Another study developed a so-called Ca^2+^/Cu^2+^ dual-ion “nano trap” for breast cancer therapy, specifically designed to counteract apoptosis evasion by inducing a combination of paraptosis and apoptosis. The nanoplatform, which encapsulates disulfiram (DSF) within amorphous calcium carbonate nanoparticles, simultaneously releases Ca^2+^, Cu^2+^, and DSF inside tumor cells. This release triggers mitochondrial Ca^2+^ overload and ROS-related dysfunction, leading to paraptosis, while toxic dithiocarbamate–copper complexes and Cu^+^-mediated Fenton-like reactions further induce apoptosis [122]. 

A recent study identified a class of small molecules capable of self-assembly that target cellular organelles such as mitochondria and enhance the immunogenicity of tumor cells. These molecules, composed of lipid conjugates and 3-(aminopropyl) triphenylphosphonium as mitochondria-targeting moieties, dynamically alter mitochondrial function, induce ER stress, and trigger cell death via both apoptosis and paraptosis [126].

### 4.5. Photodynamic Therapy (PDT)

PDT is a form of cancer treatment using light-sensitive drugs (photosensitizers) combined with specific wavelengths of light to produce ROS, causing tumor cell death, microvascular damage, and local inflammation [3]. PDT induces various cell death pathways, including paraptosis, particularly when targeting lysosomes and mitochondria to enhance photokilling [129,191,192]. PDT can induce paraptosis when applied together with agents like PyroMor (a combination of Pyropheophorbide-a and morusin), which causes the dilatation of both the ER and mitochondria [193].

Of note, most of the above-mentioned therapies do not induce paraptosis exclusively but rather trigger a combination of different cell death modalities. Observations of several different cell death characteristics are common, which is advantageous in tumor therapy as it targets multiple pathways to achieve tumor cell death.

### 4.6. Combination of Therapies

Conversely, combining different therapeutic approaches can enhance efficacy or overcome drug resistance. Recent studies highlight various combinatorial approaches to induce paraptosis in cancer cells. The proteasome inhibitor bortezomib, when combined with the Integrated Stress Response Inhibitor (ISRIB), can drive Bortezomib-insensitive breast cancer cells toward paraptosis [194]. Bortezomib, combined with nutlin-3, an effective inhibitor of the MDM2/p53 interaction, induces paraptosis in bortezomib-resistant, p53-defective solid tumor cells by causing extensive dilatation and fusion of the ER and mitochondria. This effect results from proteasomal impairment, ER stress, and disrupted Ca^2+^ homeostasis [195]. The mTOR inhibitor everolimus, combined with ginsenoside Rh2, triggers paraptosis through mechanisms such as cytoplasmic vacuolation [110]. Additionally, everolimus, combined with Jolkinolide B, enhances pro-apoptotic and pro-paraptotic effects by inhibiting Akt feedback and autophagy [165]. Jolkinolide B enhanced the efficacy of glutathione peroxidase 4 inhibitors by restoring sensitivity and potentiating both paraptosis and apoptosis, suggesting a promising combination therapy for bladder cancer [166]. Paris saponin II enhanced the effectiveness of cisplatin by utilizing a paraptosis-associated pathway [172]. Other strategies include the use of diethyldithiocarbamate and B12b in MCF-7 cells, which, in combination with short exposure to highly cytotoxic oxidized derivatives of DSF, leads to paraptosis along with lysosomal cell death [196]. 

In summary, combining different therapeutic approaches can enhance treatment efficacy and address drug resistance in cancer therapy. Studies on combination therapies indicate that integrating paraptosis-inducing agents and conventional therapies can thus significantly improve the efficacy of cancer treatment.

## 5. Conclusions

In summary, cell death is a complex and vital process essential for development, homeostasis, and disease prevention. Understanding the many different forms and the regulation of cell death, including apoptosis, necrosis, and the lesser-known paraptosis, becomes crucial for addressing disease mechanisms like cancer and neurodegeneration. Paraptosis is a form of regulated cell death characterized by ER dilatation, mitochondrial swelling, and cytoplasmic vacuolation. 

Physiological paraptosis is rare but occurs in specific contexts, such as tissue remodeling during development. Rather, paraptotic cell death is primarily observed under pathophysiological conditions as a stress response to external or internal cellular disturbances. This form of cell death is triggered by factors such as ER stress, oxidative damage, or infections, particularly in cancer, neurodegenerative diseases, and responses to toxic agents, emphasizing its role in pathological settings.

The role of paraptosis in cancer therapy is particularly promising. The growing identification of compounds capable of inducing paraptosis opens new strategies for cancer treatment by offering alternative mechanisms to bypass resistance to traditional therapies. Natural compounds have been shown to induce paraptosis by targeting pathways, such as ROS production, ER stress, and mitochondrial dysfunction. Chemical compounds also play a role in triggering paraptotic cell death, offering potent tools for selective cancer cell targeting. Moreover, combinations of different agents can potentiate anti-tumor effects, helping to overcome drug resistance by activating paraptosis alongside apoptosis. These strategies highlight the therapeutic potential of paraptosis induction in anticancer therapy, providing new and targeted therapeutic options. On the other hand, the discovery of internal cellular stressors that trigger paraptosis may help unravel its role in other diseases.

## Figures and Tables

**Table 2:** Chemical compounds inducing paraptosis in tumor cells.

Compound	Chemical Category	Type of Tumor Cell	Mechanism	Ref.
Cetylpyridinium chloride	quaternary ammonium compound	Pancreatic cancer	ER stress Accumulation of misfolded proteins Activation of MAPK	[178]
Ezetimibe	2-Azetidinone	Hepatocellular carcinoma	ER stress ROS accumulation Proteasome inhibition	[179]
Nitrovin	nitrofuran	Glioblastoma	ER stress ROS production Activation of MAPK Inhibition of thioredoxin reductase	[80]
Oxazine	heterocyclic organic compound	Breast cancer	Induction of ATF4 and CHOP Activation of JNK signaling	[177]
QR-4	isoxazolyl-urea derivative	Pancreatic cancer	ER stress Reduction of mitochondrial membrane potential Decreased Alix expression and increased levels of ATF4 and CHOP MAPK activation	[180]
QR-5	isoxazolyl-urea derivative	Colon cancer	Reduction of mitochondrial membrane potential Decreased Alix expression and increased levels of ATF4 and CHOP Suppression of Wnt/β-catenin pathway proteins	[181]
Triptycene–Peptide Hybrids	amphiphilic peptide conjugates	Cervix carcinoma Lung cancer T cell leukemia	Mitochondrial Ca^2+^ increase Membrane fusion between the ER and mitochondria	[182]

## Data Availability

The data presented in this review are openly available in [PubMed] at https://pubmed.ncbi.nlm.nih.gov, accessed on 19 September 2024.
